# Bayesian inference of spectrometric data and validation with numerical simulations of plasma sheath diagnostics of a plasma focus discharge

**DOI:** 10.1038/s41598-022-19764-7

**Published:** 2022-09-16

**Authors:** Gonzalo Avaria, Alejandro Clausse, Sergio Davis, Cristian Pavez, Nelson Villalba, Osvaldo Cuadrado, Jose Moreno, H. Marcelo Ruiz, Leopoldo Soto

**Affiliations:** 1 Research Center on the Intersection in Plasma Physics, Matter and Complexity, P2mc, Comisión Chilena de Energía Nuclear, Casilla 188-D, Santiago, Chile; 2 Departamento de Ciencias Físicas, Universidad Andres Bello, República 220, Santiago, Chile; 3 CNEA-CONICET and National University of Central Buenos Aires, Tandil, Argentina; 4 Departamento de Física, Universidad del Bio-Bio, Concepción, Chile; 5 Departamento de Física, Universidad Técnica Federico Santa María, Av. España 1680, Valparaiso, Chile

**Keywords:** Plasma physics, Optical spectroscopy

## Abstract

Plasma Foci are pulsed coaxial discharges with numerous radiation applications and interesting scientific phenomena. Although the physics answered much of the processes involved in these discharges, many related fundamental questions still remains doggedly unresolved. One of the obstacles to deeper knowledge is the scarcity of reliable experimental data. This work presents an elaborate experimental assessment of the electron density in the rundown phase of a 400 J Plasma Focus operating with hydrogen. The rundown of the plasma sheath is basically a hypersonic shock wave between two coaxial electrodes accelerated by the Lorentz force, and it is important to control the pinch formation. The electron density of the passing sheath is measured by means of the Stark broadened hydrogen alpha emission with spatial and temporal resolution. The experimental data is post-processed using Bayesian posterior probability assessment. The results are conflated with the numerical model CShock to construe an educated explanation of the sheath behavior during the rundown. In particular, it is possible to reckon the formation of a toroidal instability reported in previous experiments, and to estimate the plasma sheath temperature (4–20 eV) and velocity (62.5 km/s) at this stage.

## Introduction

Plasma Foci are pulsed coaxial discharges operating in rarefied gases. The most common setup is generally configured with two coaxial cylindrical electrodes, a central solid or hollow metallic anode and an outer barred cathode. Discharges initiate at one extreme forming a current sheet that accelerates towards the other end of the electrodes, where it collapses radially producing a Z-pinch column^1,2^ at the axis and a semispherical plasma shock perpendicular to the axis. The pinch is a bright source of pulsed emissions of x-rays^3,4^, ions^5^ and fusion products^6^, whereas the plasma shock can be used for material treatment and testing^7,8^ and, under special conditions of operation, it can be used for satellite space propulsion.

The entire discharge process can be described in six stages^9^: (i) breakdown: after the spark gap connected to the capacitor bank is triggered, a current sheath is formed above the insulator sleeve, producing the electrical breakdown of the gas present in the inter-electrode volume; (ii) rundown phase: once the current starts flowing between the electrodes, the current sheath separates from the insulator and accelerate towards the end of the coaxial electrodes; (iii) run-over: after the current sheath reaches the end of the central electrode, the current sheath collapse radially driven by the azimuthal magnetic field towards the center of the anode; (iv) pinch: close to maximum current, the current sheath is compressed into a tight plasma column where high density and temperature are achieved; (v) plasma disruption: due to magnetic instabilities present in these type of plasmas, the plasma column is disrupted and a highly energetic plasma shock is expelled perpendicular to the axis^7^; and (vi) plasma jets: a few hundreds of nanoseconds after the column disruption a plasma jet is often observed^10^.

Although the physics answered much of the described stages, many related fundamental questions still remains doggedly unresolved. One of the main obstacles to deeper knowledge is the scarcity of reliable experimental data of the local instantaneous state of the plasma sheath, partly due to the experimental difficulties involved in the diagnostics of such short pulsed plasmas, about tens of ns, and the extreme thermodynamic conditions reached in the plasma. In the present work we focus our attention on the rundown phase, which basically is a hypersonic shock wave accelerated by the Lorentz force. The plasma shock hauled along the axis direction has been proposed for several applications, like surface ion implantation^11^, and, recently, it was shown that the final blast energy is enough to experimentally recreate the conditions necessary for magnetic-fusion material testing^8,12^. Therefore, any further knowledge that can be obtained from experimental techniques is of great value. For all the mentioned applications, the evolution of the thermodynamic state of the plasma sheath is a crucial information for design and management.

The plasma focus has the special feature that is a self-scale kind of z-pinch^13,14^. For devices in the range 1 MJ–0.1 J of stored energy^15,16^ optimized for neutron emission, the axial rundown velocity and radial compression velocity has practically the same value, independent of the stored energy, being $4 \times 10^{4}$–$1 \times 10^{5}$ m/s and $1 \times 10^{5}$–$2 \times 10^{5}$ m/s respectively; the same ion plasma density in the pinch in the order of $10^{24}$–$10^{25}\,\mathrm{m}^{-3}$, same magnetic field in the pinch edge of order of 10–20 T, same Alfvén speed estimated to be above $1 \times 10^{5}$ m/s, and same temperature $\sim$ 0.5–10 keV^13^. However, the plasma stability depends on the size and energy of the device. Therefore, the study in a plasma focus device working in a particular energy, contributes to the understanding of the common physics related to plasma focus^14^.

Several studies have been presented in connection with the impact of the rundown and the pinch formation providing preliminary insights of the sheath characteristics: Zakaullah et al.^17^ used a $\beta$-source at the bottom of the insulator in order to improve neutron production by enhancing the ionization during breakdown of a Plasma Focus discharge; Veloso et al.^18^ compared the detachment time and plasma sheath thickness with the neutron emission in a 400J Plasma Focus discharge; Barbaglia et al.^19^, considered different cathode configurations on the PACO device (1.9 kJ) in order to study the influence of the geometrical dimensions to the neutron production and also its effect on the drive parameter. Ultrafast photographic visualization and electrical signal processing are amongst the popular techniques used to measure the shape and velocity of the sheath. Veloso et al.^20^ measured the rundown velocity of the plasma sheath in the same device used in the present work, by means of an array of optical fibers connected to photodiodes. With this setup, an average rundown velocity of $4 \times 10^{4}$ m/s was determined. Using a similar setup, which consisted of two fiber arrays focused in the inner and outer edges of the inter-electrode volume, Caballero-Bendixen et al.^21^ reported velocities of $10^{5}$ m/s and sheath masses between 1 and $6\,\upmu$g, with a dragged mass fraction of 7–5% on a 2 kJ dense plasma focus operating with Ne gas. The same order of velocities were reported by Tauschwitz^22^ using Schlieren diagnostics in a 1 kJ Plasma Focus. Interferometric techniques were used to measure the electron density of the pinch in several devices^23,24^. Validated numerical models based in the snowplow scenario are currently available to assist in the interpretation of the experimental data from the point of view of physical conservation principles. In particular, in the present work we exploit the 2D computer code CShock, which has shown excellent capabilities to simulate the rundown phase^25–27^.

Interferometry is a known reliable technique in plasma diagnostics, and it has been applied successfully in Plasma Focus devices to determine the plasma density at the pinch phase^23,24^. A significant problem of using interferometric techniques to diagnose the plasma sheath in the rundown phase is the detection limit due to the diffraction effect, imposing a lower bound on the detectable electron density of about $10^{23}\,\mathrm{m}^{-3}$ for a pinch size of around 1 mm. This limitation complicates the measurements at the early stages of the discharge, when the plasma density is still low. A possible workaround is to use visible spectroscopy and Stark broadening to measure the electron density in the plasma sheath. In a pioneer work, Feugeas et al.^28^ measured the evolution of the line emission intensity of the plasma stream in a 1-kJ Plasma Focus. Later, several experiments were conducted to measure the electron density and temperature of the plasma sheath on different experimental configurations^29–31^. A comprehensive work has studied the electron density and temperature of the plasma in the high-energy device $PF-1000$, including the interaction with tungsten targets^32–35^. The range of values reported so far are densities between $10^{22}$ and $10^{24}\,\mathrm{m}^{-3}$, and temperatures between 3 and 5 eV.

The present work reports a study following a similar approach, in combination with posterior Bayesian processing, to determine the most probable electron density distributions of the plasma sheath, responsible for the observed spectra. Spatially and temporally resolved spectra of the alpha emission of hydrogen was acquired in a middle point between electrodes along the rundown passage of a 400J Plasma Focus. The electron density was estimated from the Stark broadened emission. The results are finally compared with numerical simulations to produce a physically based explanation of the phenomena involved in the rundown phase.

## Results

**Figure 1:**
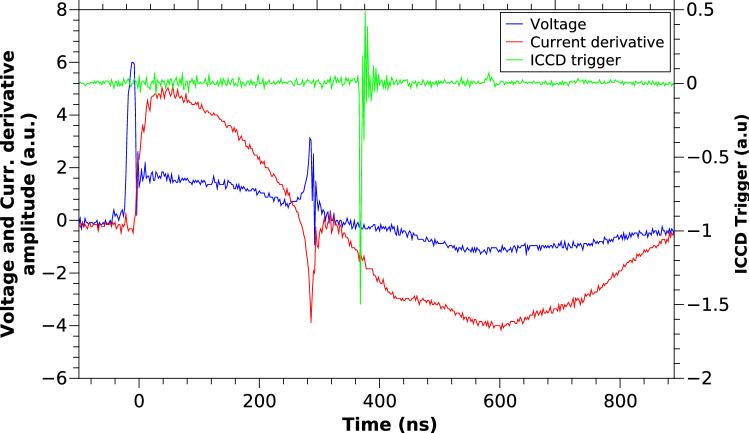
Discharge voltage (a.u.), current derivative (a.u.) and ICCD monitor pulse (a.u.) for a typical discharge pulse. The dip seen in the current derivative signal is associated to the column compression moment. The operating pressure was 9 mbar of hydrogen.

Figure 1 shows typical electrical signals that describe the evolution of the current sheath on a plasma focus discharge. The first peak seen in the discharge voltage signal is associated to the electrical breakdown on the surface of the insulator. After breakdown has been achieved, the current starts to flow, represented by the rapid increase on the current derivative signal. Later, the current sheath starts to move through the electrodes until it reaches the top of the anode and it starts to move towards the center of the electrode, where the plasma column is formed and compressed. This compression is seen in the current derivative signal as a dip, around 300 ns. After that, the plasma column is disrupted and continues to move in the axial direction^7^. The third electrical signal corresponds to the Intensified Capacitively Coupled Device (ICCD) trigger pulse, indicating when the spectral image was acquired.

**Figure 2:**
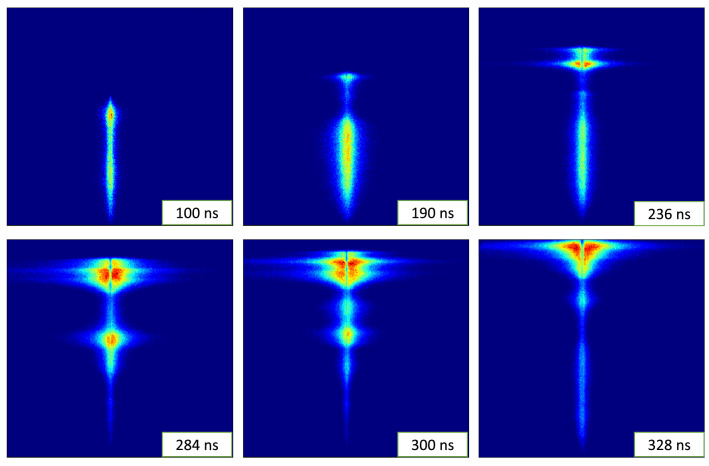
Time sequence of the spectral images for the discharge. At earlier times it can be seen that a plasma is formed at the inter-electrode volume, which later starts to move towards the top of the electrodes.

**Figure 3:**
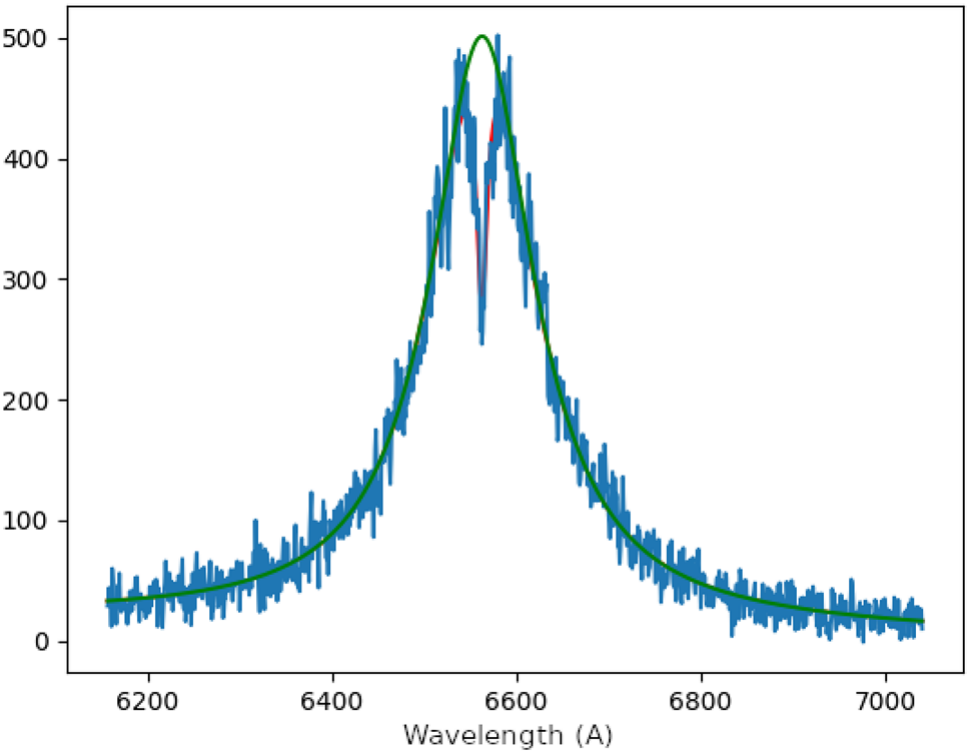
Intensity profile for the H-alpha emission, showing the fitted curve following the model proposed by Kielkopf^38^ (red) and the opacity corrected profile (green) which is used for the electron density calculations.

**Figure 4:**
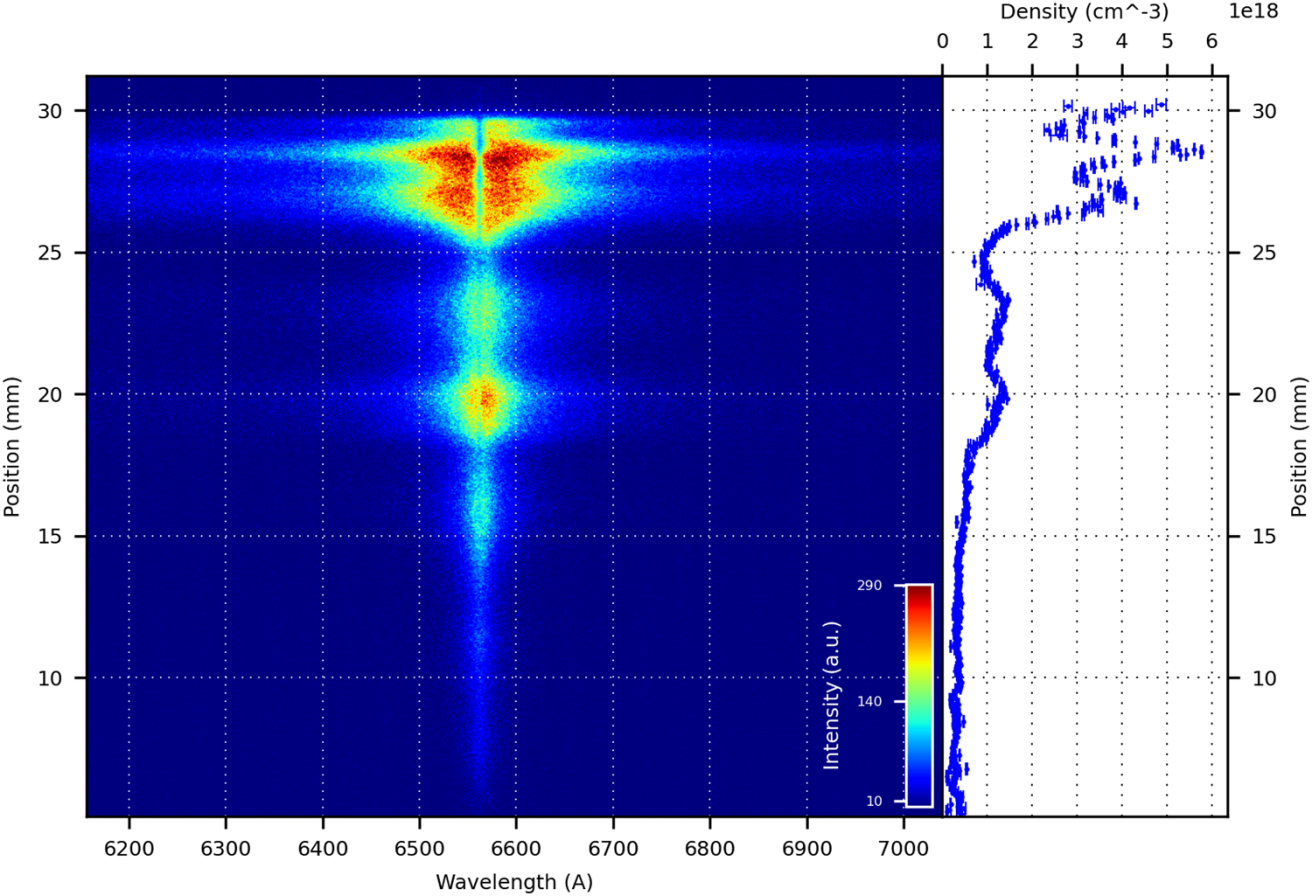
Spectral image of the inter-electrode space, 300 ns after the onset of the current. As seen from the density profile, the electron density is around $6 \times 10^{18}\,\mathrm{cm}^{-3}$ at approximately 28 mm from the bottom of the electrodes.

Figure 2 shows a sequence of images taken at increasing times, namely 100, 190, 236, 284, 300 and 328 ns. All the times are measured assuming by convention *t* = 0 the moment when the temporal derivative of the current starts rising and crosses 50% of its maximum value. The vertical direction corresponds to the axial coordinate parallel to the electrodes axis. The current sheet moves upwards. The horizontal coordinate of the images corresponds to the wavelength, centered at 6562 Å ($H_{\alpha }$) and an approximate bandpass of 88 nm. Since the electron density is proportional to the width of the $H_{\alpha }$ line, the broader emission represent denser plasma^36,37^. The opacity of the plasma is seen as a dip in intensity at the center of the emission line. It has been accounted in the line width calculations following the method proposed by Kielkopf^38^. A self-absorption corrected spectrum can be seen in Fig. 3. The spectral images in Fig. 2 show that most images present a frontal bright spot followed by a sort of tail. The electron density associated with the position of the emission in the inter-electrode space can be seen in Fig. 4, and was calculated from the value of 2*w* estimated from the Bayesian posterior probability calculations of the parameters that define the curve in Eq. (7).

To get a sensible interpretation of the images and glean useful information out of them, we perform numerical calculations of the evolution of the current sheet using the code CShock^26,27^, which is based in a 2D model that was verified with several experiments, working particularly well in tracking the shaping of the current sheet^25,39^.

**Figure 5:**
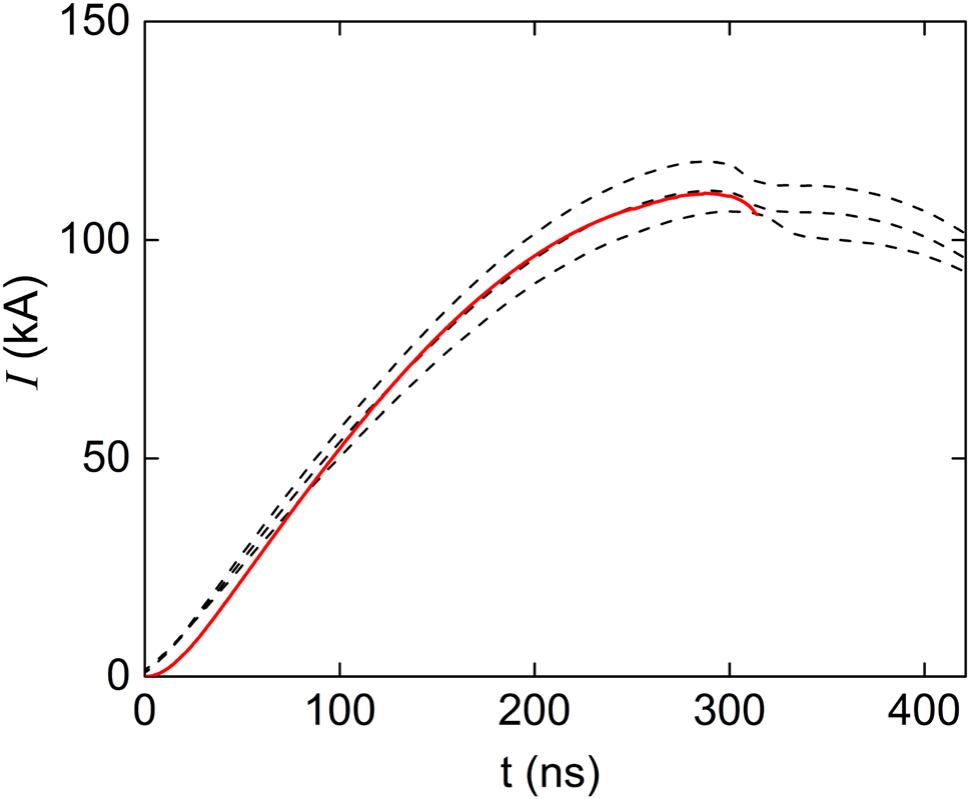
Experimentally measured current (dashed line) compared to the numerically calculated current by means of the CShock code. The operating pressure was 9 mbar of hydrogen.

Figure 5 compares the evolution of the current calculated numerically with the current signals of three of the discharges with the same experimental conditions. The breakup parameter was set in $\alpha = 95\,\upmu \mathrm{s}^{-1}$, which amounts to a breakup delay of 10.5 ns^26^. The discretization of the current sheet was set in 2 nodes/mm, the time step 1 ns and the initial mass was $2\times 10^{-9}$ kg.

**Figure 6:**
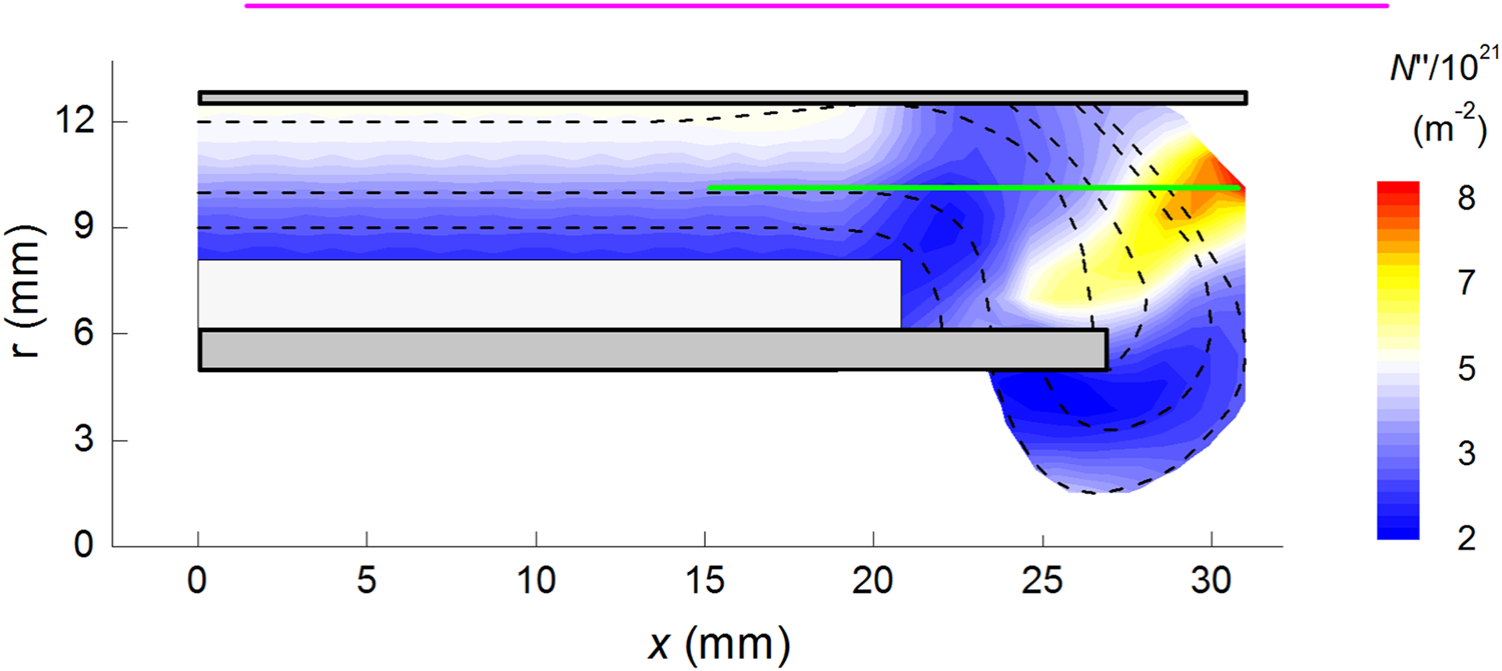
Color map of the numerical calculation of the density per unit of frontal area of the current sheet. The dashed curves indicate the sequence of shapes that the current sheet takes during the discharge starting at lift off from the insulator up to the beginning of the pinch compression. The color of each point stands for the proton surface density that the sheet has when it passed through that point, i.e., the number of protons contained in a unit of frontal area of the current sheet. The green line indicates the area that is imaged into the spectrometer. The simulation was performed with the code CShock, which calculates the evolution of plasma sheath up to the instant when the front touches the axis. The pinch compression is not included in the calculations.

Figure 6 shows a sequence of dashed curves representing the evolution of the shape of the current-sheet during a discharge, starting at lift off from the insulator up to the beginning of the pinch compression. The color of each spatial point (*x*, *r*) stands for the proton surface density that the sheet has when it passed through that point, i.e., the number of protons contained in a unit of frontal area of the current sheet. The green line in Fig. 6 represents the axial coordinate of the images. The estimated position of the current sheet is given by the intersection of the green line with dashed curve corresponding to the time each image was acquired. Tracking this position for all the images, it was possible to conclude that the current sheet corresponds to the frontal bright spot of each image. Moreover, the numerical calculations show that when the current sheet first intersects the area imaged into the spectrometer, it is parallel to the line. This event occurs at about 190 ns. In Fig. 2 it can be seen that the image corresponding to 190 ns indeed presents a bright elongated region, which can be safely interpreted as the first illumination of the current sheet in its radially expansion from the insulator towards the cathode. Afterwards, the numerical simulation shows that the current sheet intersects the observation line transversally, running down towards the anode end. The position of the calculated intersection is consistent with the uppermost bright spot observed in the images recorded at approximately 200 ns and thereafter.

**Figure 7:**
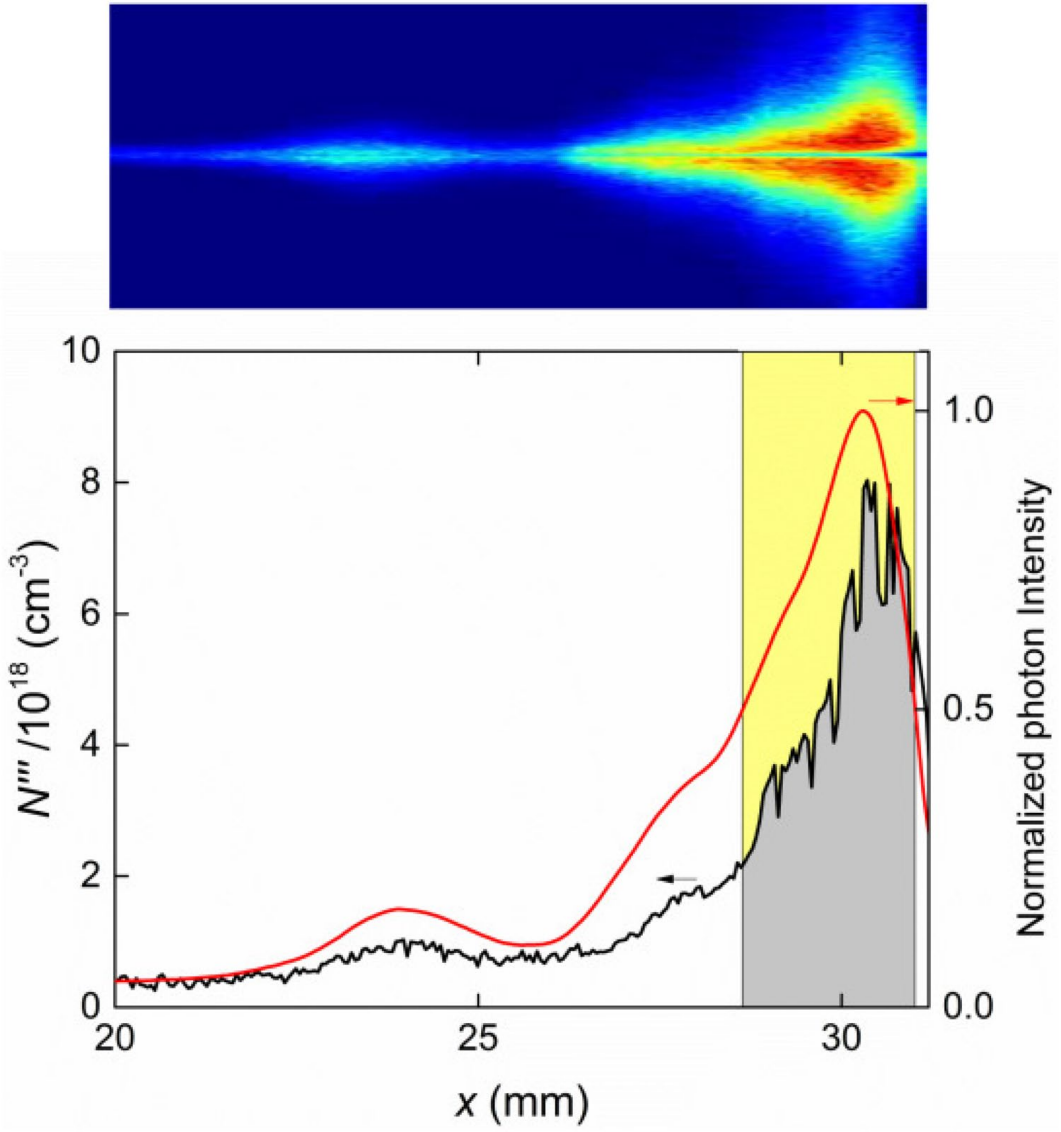
Image processing method for estimating the electronic density per unit of sheet area. The graphic shows the profiles of photon intensity and electronic density. The current sheet is identified with the shadowed region below the density profile, whose limits are defined by the positions on both sides of the peak intensity where the intensity is half the one at the peak. The shadowed area is then the electronic density per unit of frontal area of the sheet.

Figure 7 shows one of the images together with the corresponding electronic density and ICCD intensity counts along the observation linear area at the inter-electrode space. Assuming total ionization of the hydrogen atoms, the electronic density equals the proton density. The proton density per unit of frontal area of the sheet can be then estimated by integrating the electronic density profile within the sheet thickness. Now, the exact position of the frontal and back boundaries of the sheet is not obvious from the images. Nevertheless, a reference to assess these boundaries can be given by the photon intensity, for it can be safely ascribed to the ionization process. Accordingly, we propose to integrate the electronic density in the region where the photon intensity exceeds 50% of the peak intensity. Figure 7 depicts the procedure. The superficial density is the shadowed area of the plot, which formally is defined as:

1 $$\begin{aligned} N^{\prime \prime } = \int _{x_{-}}^{x_{+}} N^{\prime \prime \prime } dx \end{aligned}$$

where $x_{-}$ and $x_{+}$ are the positions where the photon intensity *I* satisfy:

2 $$\begin{aligned} I(x_{\pm }) = \frac{1}{2} I_{max} \end{aligned}$$

and the subindexes − and + indicate the left and right side respect to the peak.

**Figure 8:**
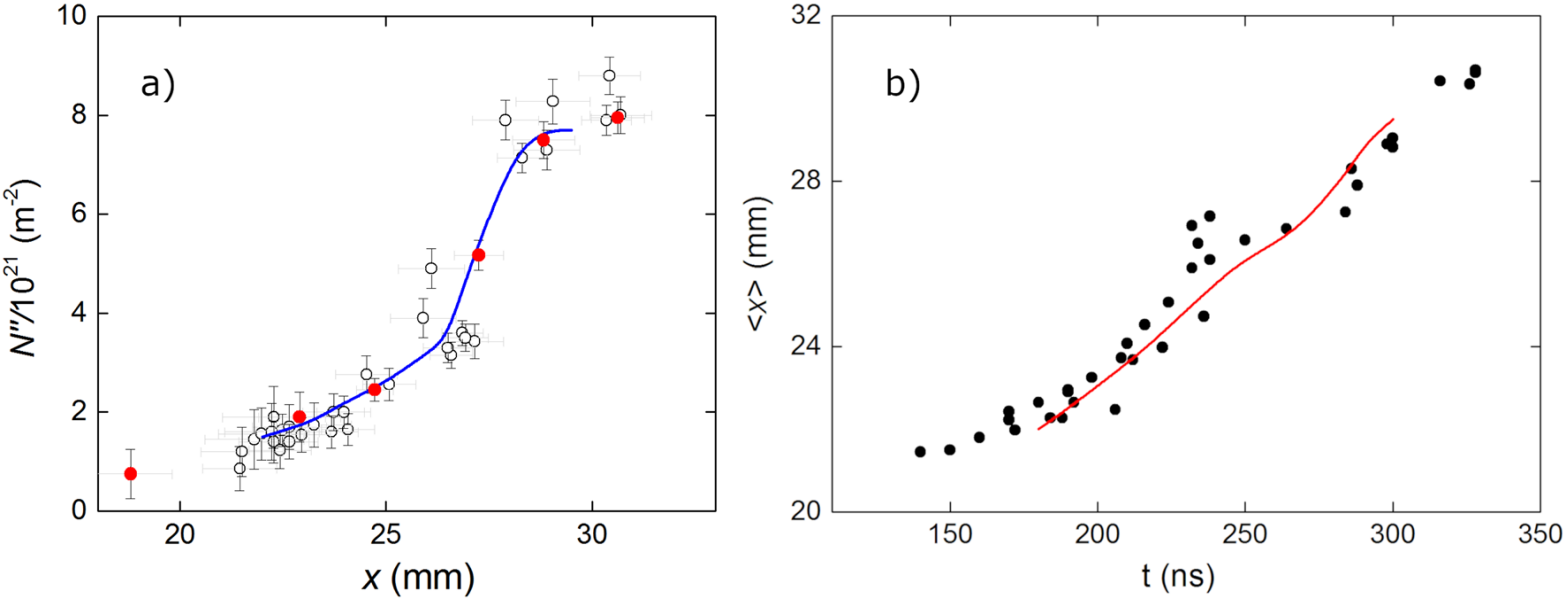
(**a**) Estimated electronic density per unit of frontal area of the sheet, plotted against the position of the center of the current sheet, $x = (x_{-}+x_{+})/2$. The horizontal bars represent the estimated boundaries of the sheet. The vertical bars are uncertainties estimated from the noise of the profiles. The red symbols correspond to the images shown in Fig. 2. (**b**) Temporal evolution of the position of the current sheet, numerical (curve) and experimental (symbols). An axial phase speed of $\sim 6.25 \times 10^{4}$ m/s can be estimated from the experimental observations.

Figure 8a depicts the resulting values of $N''$ plotted against the position of the center of the integration range, that is:

3 $$\begin{aligned} \langle x \rangle = \frac{1}{2}(x_{-}+x_{+}) \end{aligned}$$

The solid curve shown in the graphic is the numerical calculation of $N''$, which corresponds with the succession of background colors in Fig. 6 along the focal line (green). Figure 8b shows the temporal evolution of $\langle x \rangle$. The average velocity is $6.25 \times 10^{4}$ m/s. The curve in the graphic is the corresponding numerical calculation.

Some interesting features can be distinguished in Figs. 2, 3, 5, 6, 8, namely:

- The sheet superficial density, $N''$, along the focal line increases as the shock advance, even during the run over stage where the sheet surface substantially increases.
- Up to $x = 26$ mm, $N''$ increases steadily at an average rate of $0.4\times 10^{21}\,\mathrm{m}^{-2}$ per axial mm. This is a worthy result, for, assuming a planar snowplow model, the number of electrons swept by the piston per mm of rundown is
  4 $$\begin{aligned} \frac{2p}{kT} = 0.47 \times 10^{21}\,{\rm m}^{-2}/{\rm mm} \end{aligned}$$
  Therefore, the corresponding snowplow rundown coefficient is estimated as 0.85, and it can be reasonable been ascribed to a shape effect.
- From *x* = 26.5 mm to about *x* = 29 mm, there is a sharp increase of $N''$. The average growth rate during this stage is about $2.3\times 10^{21}\,\mathrm{m}^{-2}/\mathrm{mm}$, almost 5 times that of the first stage. To interpret this effect, we should analyze the color map of $N''$ shown in Fig. 6. It can be seen that this sudden increase coincides with the line of sight intersecting a mass clutter, which originates in the corner at the insulator foot, and moves upward describing a convex curved trajectory. This particular feature has been observed in the $PF-400J$ discharge by Schlieren diagnostics^7,40^, as well as in the $PF-50J$ discharge^41^ by means of interferometry measurements^39^. The cause of the mass cluster is evinced by realizing that the volume swept by that part of the sheet is larger than other points of the same surface.
- The data for larger values of *x* correspond to the run over stage and radial collapse of the current sheet. The spectrometer is observing only a small region of the inter-electrode volume, thus only able to observe a small part of the sheet at *r* = 10 mm, so there is actually only indirect information about what happens in the parts of the sheet moving towards the pinch. The registered values of $N''$ are produced by the path of the mass clutter mentioned in the last paragraph.

**Figure 9:**
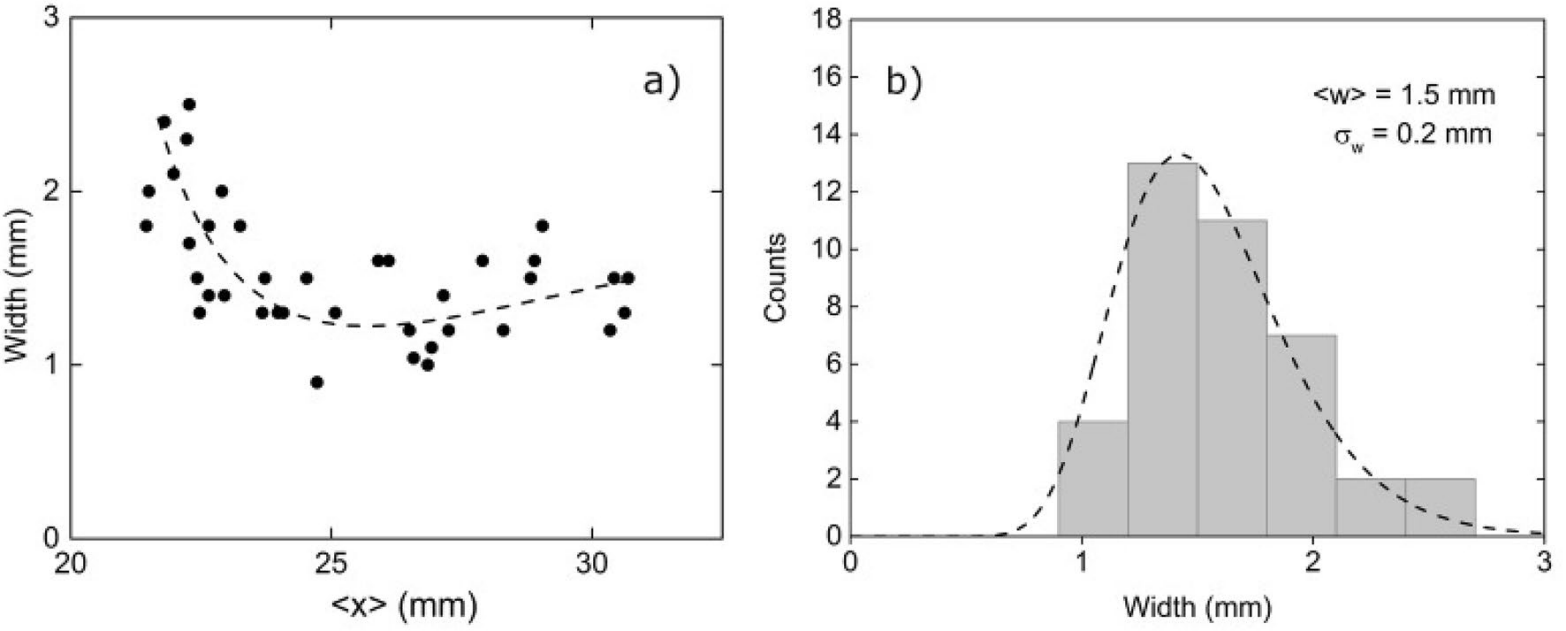
(**a**) Estimated width of the current sheet at the intersection with the focal line. (**b**) Histogram of the recorded width of the current sheet at the intersection with the focal line.

Figure 9a shows the thickness of the plasma sheet at the moment and place where it encounters the focal plane, as resulted from the image processing method described above, that is, the length of the segment of the image where the intensity is higher than half the peak intensity, that is:

5 $$\begin{aligned} w = x_{+}-x_{-} \end{aligned}$$

Up to $x\sim 25$ mm, the width decreases from approximately 2.5 mm to approximately 1.2 mm. Thereafter, the width increases slightly to approximately 1.5 mm. Nevertheless, since the data dispersion is about 1 mm, this information should be handled with care in drawing conclusions from them. The mean width is 1.5 mm, with a standard deviation of 0.2 mm (see Fig.9b).

Using the information from Figs. 8 and 9, it is possible to estimate the average electronic density of the plasma sheet at the moment and place where it encounters the laser beam, that is:

6 $$\begin{aligned} \langle N''' \rangle = \frac{N''}{w} \end{aligned}$$

**Figure 10:**
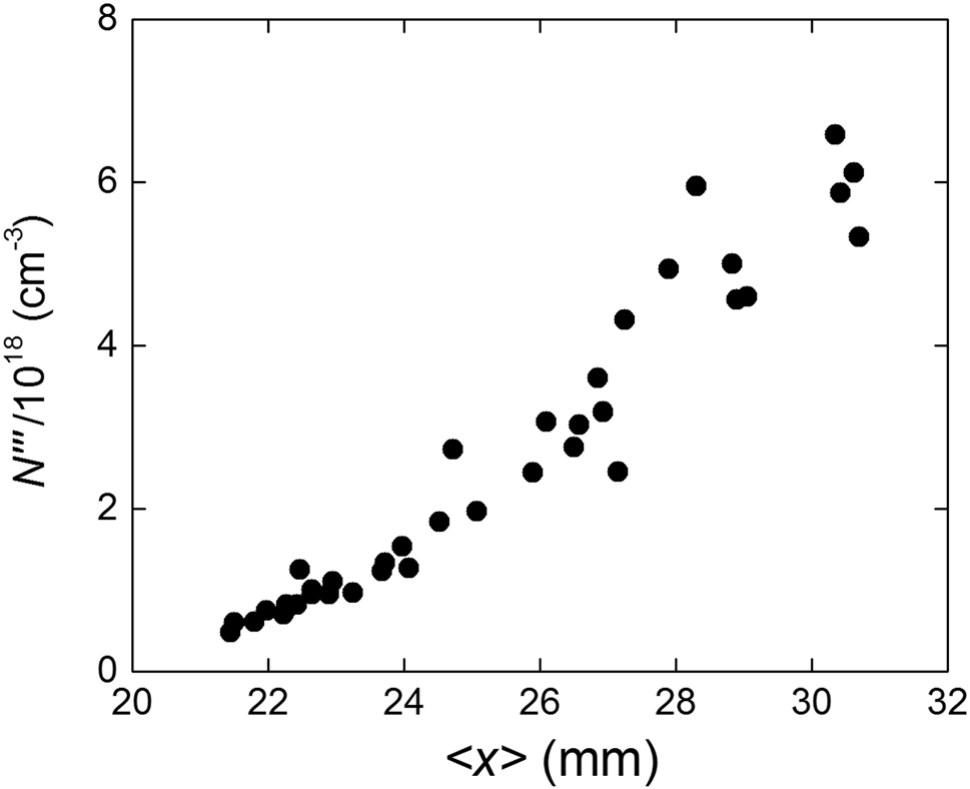
Evolution of the average electronic density at the intersection with the focal line.

Figure 10 shows the dependence of $\langle N^{\prime \prime \prime } \rangle$ on the sheet position $\langle x \rangle$.

## Discussion

This work presents spectroscopic measurements of the axial acceleration phase on a Plasma Focus device, and the comparison of the inferred results with a two dimensional numerical model. The electron density was estimated from the opacity corrected broadening of the hydrogen-$\alpha$ emission, where the uncertainty of the spectral width of the emission line was computed with a Bayesian posterior probability calculation, using Markov Chain Monte Carlo sampling.

A plasma sheath speed of $\sim 6.25 \times 10^{4}$ m/s can be estimated for the axial acceleration phase, from the slope of Fig. 8b. The estimated electron density for the axial acceleration phase ranged between $0.5 \times 10^{18}\,\mathrm{cm}^{-3}$, for earlier times in the current pulse evolution, to $8.0 \times 10^{18}\,\mathrm{cm}^{-3}$ at the moment of maximum current intensity (when the axial phase is finished and the radial phase begins) for a temperature of $T \sim 4{-}20$ eV.

As seen from the comparison of the numerical model (CShock) with the measured density profiles at different times in the current pulse evolution, several interesting features were identified: the superficial density of the plasma sheath increases with position; an increase in the growth rate of the plasma sheath can be attributed to the appearance of a previously observed mass clutter that is formed at the intersection of the insulator and the anode. As can be seen from the comparison of the measured densities and the calculated parameters, a good agreement between the numerical model and the experimental measurements is achieved.

## Methods

### Experimental setup

The spectroscopic measurements were performed in the low energy plasma focus device PF-400J (176–539 J, 850 nF, 20–35 kV, quarter period 300 ns) ^6^. The device was operated in Hydrogen at pressures ranging from 9 to 15 mbar at a repetitive scheme (0.06 Hz) with a charging voltage of $\sim 27$ kV ($\sim 310$ J). The anode is a stainless steel electrode of 12 mm outer diameter, 10 mm inner diameter, with an effective length (distance between the insulator sleeve and anode top) of 6.5 mm. The cathode is composed of eight 5.0 mm diameter stainless steel rods, placed at a distance of 12.5 mm from the center of the anode.

**Figure 11:**
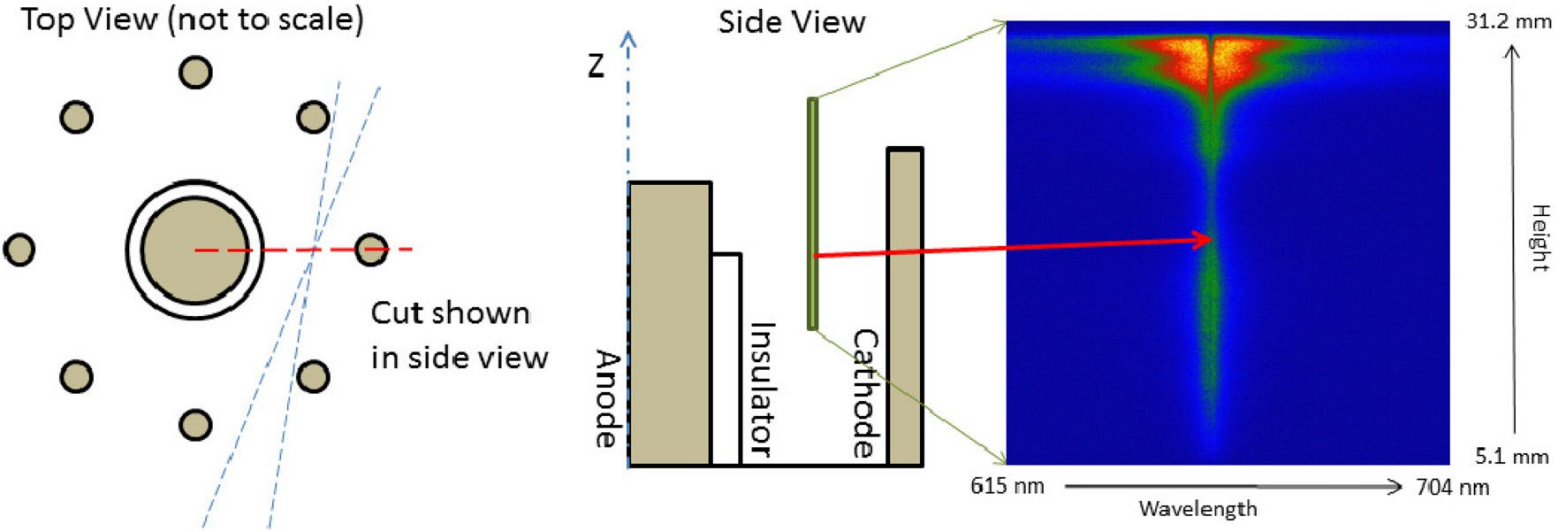
Scheme of the focus plane inside the inter-electrode volume and the representation of the strip that is imaged at the spectrometer slit, which produces a spectral image of the plasma sheath during an instant of the discharge. The spatial resolution is given by the magnification of the system.

The spectra were acquired with an imaging 0.5 m focal length Czerny-Turner spectrometer (Shamrock 500i) coupled to a $1024 \times 1024$ pixel ICCD detector (iStar 334). A 600 groove/mm diffraction grating was selected considering the bandpass and efficiency at the wavelength range under study. In order to obtain the spatial resolution of the light emitted from the inter-electrode region, a *f* = 200 mm fused silica plano-convex lens was used to form an image of a small region of the plasma at the spectrometer entrance slit(30 $\upmu$m). A magnification of $\sim 0.5 \times$ enabled the detection of the light emitted from a rectangular plasma region $\sim 26$ mm high and $\sim 60\,\upmu \mathrm{m}$ wide (Fig. 11). This region was located at 10.3 mm from the discharge axis and at a height of 5.1 mm from the cathode plate. The observation plane was selected to be between the central electrode and one of the cathode bars as seen on the Fig. 11.

The ICCD-Spark gap system was synchronized externally, allowing the acquisition of time resolved measurements with integration times of 3 ns at any time along the current pulse. The high reproducibility of the discharge allowed the description of the evolution of the plasma in time by collecting single spectral images in each shot (Fig. 11), producing the temporal sequence of the alpha emission peak, corresponding to the passage of the plasma sheath at different times of the rundown stage. As seen from Fig. 11, the spectral image is defined by a “Wavelength” horizontal axis and a “Position” vertical axis. This spectral image has information of the emission spectra generated from the different positions along the vertical axis. To obtain a spectrum at a specific position, the intensity profile can be extracted from that position by vertically binning a small number of horizontal lines from the image.

In order to analyse the spectral images, the position of the plasma sheath was defined at the 50% of the maximum intensity of each spectral image, measured from the top of the electrodes towards the bottom (i.e., from the top of the image). By plotting the plasma sheath position versus the time of the acquired image, the plasma sheath velocity was estimated to be $62.5 \pm 0.8$ km/s.

### Electron density measurements

Electron density measurements of the plasma sheath were acquired from the Stark broadened hydrogen-$\alpha$ emission at 6562.8 Å ^36,37^. To obtain a good signal-to-noise ratio, each spectral image was divided into stripes, 10 pixels wide (equivalent to $\sim 250\,\upmu \mathrm{m}$) by 1024 pixels long (wavelength), that were overlapped by 5 pixels. An intensity profile was obtained by binning the individual stripes, i.e. a single intensity profile is obtained by adding the intensities of ten rows of $1\,\mathrm{pixel}\,\times \,1024\,\mathrm{pixel}$ size.

In order to account for the opacity broadening and self-absorption seen in the $H_{\alpha }$ emission, the expression proposed by Kielkopf et al.^38^ was fitted to the intensity profiles, after the wavelength calibration had been performed:

7 $$\begin{aligned} F(\lambda ) = f\frac{w^{2}}{(\lambda - \lambda _{0} - d)^{2}+w^{2}} \times \exp \left( -a\frac{w^{2}_{a}}{(\lambda - \lambda _{0} - d_{a})^{2} + w^{2}_{a}} \right) + b + c(\lambda - \lambda _{0}) \end{aligned}$$

where 2*w* is the Full Width at Half Maximum (FWHM) of the $H_{\alpha }$ emission centered at $\lambda _{0} = 6562.8$ Å, $2w_{a}$ is the FWHM of the absorption peak and $d,d_{a}$ are the line shifts of the $H_{\alpha }$ line and the absorption peak. The electron density $N^{\prime \prime \prime }$ was calculated by using equation (2) as in Ref. ^38^:

8 $$\begin{aligned} 2w = 1.55 \times 10^{-11} (N^{\prime \prime \prime })^{0.70 \pm 0.03} \end{aligned}$$

considering the estimated 2*w* value obtained from the Bayesian posterior probability of the parameters, explained in detail below.

The curve in Eq. (7) was fitted to the intensity profiles using the nonlinear least squares method, as implemented in the *LMFIT Python library* ^42^. Since the spectral images are unique, the line profiles obtained are also unique for each acquisition time. In order to estimate the uncertainty of the fitted parameters obtained from the nonlinear least squares method given the measured data, we have computed the Bayesian posterior probability ^43,44^ of these parameters using Markov Chain Monte Carlo sampling ^45^, as implemented in the *EMCEE Python library* ^46^. Unlike traditional (frequentist) statistics, where the model is fixed beforehand and uncertainty is understood as reflecting all the possible hypothetical measurements that one could perform in the future (but have not been made), Bayesian statistics deals with all the possible models (or parameters of a fixed model) given the actual measurements performed, by assigning each model or parameter a *posterior probability* after the data is taken into account. In our case, if we write our model $F(\lambda )$ as $F(\lambda ; \varvec{\theta })$ where $\varvec{\theta }=(w, w_a, d, d_a, f, a, b, c)$ is the set of parameters, and denote our measured data by *D*, then we have by Bayes’ theorem,

9 $$\begin{aligned} P(\varvec{\theta }|D, I_0) = \frac{P(\varvec{\theta }|I_0)P(D|\varvec{\theta }, I_0)}{P(D|I_0)}, \end{aligned}$$

where $I_0$ is the prior state of knowledge, $P(\varvec{\theta }|D, I_0)$ is the posterior distribution of parameters given the data, $P(\varvec{\theta }|I_0)$ is the prior distribution of parameters and $P(D|\varvec{\theta }, I_0)$ is the *likelihood function*. In the case where *D* consists of *n* independent measurements

$$\begin{aligned} D=\{(F_1,\lambda _1), (F_2, \lambda _2), \ldots , (F_n, \lambda _n)\} \end{aligned}$$

and the nonlinear least square method is used, the likelihood function becomes a product of Gaussian distributions,

10 $$\begin{aligned} P(D|\varvec{\theta }, I_0) = \prod _{i=1}^n \frac{1}{\sqrt{2\pi }\sigma }\exp \Big (-\frac{1}{2\sigma ^2}(F_i - F(\lambda _i; \varvec{\theta }))^2\Big ). \end{aligned}$$

### Numerical modeling of the plasma-sheath dynamics

The evolution of the current sheet (CS) was modeled by means of CShock ^27^, which is a validated numerical code of PF discharges based in a two-dimensional model. The CS is represented by a set of moving coaxial conical elements obeying the conservation equations of mass, momentum and energy. Each CS element is represented by variables accounting for the mechanical state of the plasma, namely mass, position, velocity, acceleration and density. The evolution of the mass (*m*) and momentum (*mv*) of a given CS element of frontal area *A*, length *l*, velocity *v*, located at radius *r*, satisfy the following conservation equations:

11 $$\begin{aligned}&\frac{dm}{dt} = \rho _{0} A v \end{aligned}$$

12 $$\begin{aligned}&\frac{d(mv)}{dt} = \frac{\mu _{0}}{4\pi }\frac{l}{r}I^{2} \end{aligned}$$

where $\rho _{0}$ is the density of the stagnant gas, $\mu _{0}$ is the vacuum permeability and *I* is the instantaneous electrical current, which is calculated from the equation of an electrical circuit with variable inductance, that is:

13 $$\begin{aligned}&\frac{d}{dt}\left[ (L_{ext}+L_{g}) I \right] + \frac{Q}{C} = V_{sg} \end{aligned}$$

14 $$\begin{aligned}&\frac{dQ}{dt} = I \end{aligned}$$

where *Q* is the charge in the condenser bank, *C* is the bank capacity, $L_{ext}$ is the inductance of the external circuit, $L_{g}$ is the inductance of the gun and the CS, and $V_{sg}$ is the combined voltage drop in the spark gap and the gas between the electrodes during the breakdown. This voltage drop is modelled as

15 $$\begin{aligned} V_{sg}(t) = V_{0}\frac{1+e^{\alpha t_{0}}}{1+e^{\alpha (t-t_0)}} \end{aligned}$$

where $\alpha$ and $t_{0}$ are characteristics parameters of the closing properties of the spark gap and electrodes system, which should be calibrated with the electrical signals.

Each CS element moves perpendicular to its frontal area with velocity *v* calculated with Eqs. (11) and (12). The number of segments representing the CS is not fixed but changes with time. This introduces some difficulties in carrying out the simulation because the elements separate from each other at each time step. In order to keep the coherence the CS should be reconstructed at each step, which must be done properly to avoid numerical instabilities. This problem was successfully solved using the techniques discussed in Casanova et al.^26^. Once a CS segment reaches the position of the outer electrode radius, it is assumed that the current flows through the outer electrode and the element is no longer considered part of the CS. The inductance $L_{g}$ is calculated by approximating the contribution of each CS element as a small coaxial cylinder. It should be stressed that CShock does not require the introduction of sweeping parameters since, unlike the planar pistons models^47–49^, the shape of the current sheet is solved along with the evolution of the electrical variables. The introduction of sweeping parameters is generally needed in planar piston models to compensate for the fact that the shape of the sheath is not planar and for the mass expulsion between the cathode bars. These two effects are taken into account by CShock. The only parameters required to calibrate the simulation are those associated with the breakdown delay.

## Data Availability

The datasets used and/or analysed during the current study available from the corresponding author on reasonable request.
